# Deletion of the Alzheimer’s disease risk gene *Abi3* locus results in obesity and systemic metabolic disruption in mice

**DOI:** 10.3389/fnagi.2022.1035572

**Published:** 2022-12-22

**Authors:** Daniel C. Smith, Hande Karahan, H. R. Sagara Wijeratne, Mamun Al-Amin, Brianne McCord, Younghye Moon, Jungsu Kim

**Affiliations:** ^1^Stark Neurosciences Research Institute, Indiana University School of Medicine, Indianapolis, IN, United States; ^2^Medical Scientist Training Program, Indiana University School of Medicine, Indianapolis, IN, United States; ^3^Medical Neuroscience Graduate Program, Indiana University School of Medicine, Indianapolis, IN, United States; ^4^Department of Medical and Molecular Genetics, Indiana University School of Medicine, Indianapolis, IN, United States; ^5^Department of Biochemistry and Molecular Biology, Indiana University School of Medicine, Indianapolis, IN, United States

**Keywords:** obesity, microglia, *Abi3*, hypothalamus, Alzheimer’s disease

## Abstract

Alzheimer’s disease (AD) genetics studies have identified a coding variant within *ABI3* gene that increases the risk of developing AD. Recently, we demonstrated that deletion of the *Abi3* gene locus dramatically exacerbates AD neuropathology in a transgenic mouse model of amyloidosis. In the course of this AD project, we unexpectedly found that deletion of the *Abi3* gene locus resulted in a dramatic obese phenotype in non-transgenic mice. Here, we report our investigation into this serendipitous metabolic finding. Specifically, we demonstrate that mice with deletion of the *Abi3* gene locus (*Abi3^–/–^*) have dramatically increased body weight and body fat. Further, we determined that *Abi3^–/–^* mice have impaired energy expenditure. Additionally, we found that deletion of the *Abi3* gene locus altered gene expression within the hypothalamus, particularly within immune-related pathways. Subsequent immunohistological analysis of the central nervous system (CNS) revealed that microglia number and area were decreased specifically within the mediobasal hypothalamus of *Abi3^–/–^* mice. Altogether, this investigation establishes the functional importance of the *Abi3* gene locus in the regulation of systemic metabolism and maintenance of healthy body weight. While our previous findings indicated the importance of *Abi3* in neurodegeneration, this study indicates that *Abi3* related functions are also essential for metabolic regulation.

## 1 Introduction

Perturbations in immune function have been demonstrated to exacerbate neurodegeneration and metabolic diseases (Lumeng and Saltiel, 2011; Ransohoff, 2016; Reilly and Saltiel, 2017). Additionally, it has been reported that metabolic diseases, such as obesity, increase the risk of developing neurodegenerative disease such as Alzheimer’s disease (AD) (Mazon et al., 2017; Zhuang et al., 2021). Intriguingly, it has been suggested that disrupted immune function is a potential link between these disease states (Pugazhenthi et al., 2017; Newcombe et al., 2018). However, the field currently has limited knowledge regarding the specific molecular drivers that alter the immune system to jointly modulate metabolic and neurodegenerative disease states. In this report, we describe how an unexpected discovery revealed that an AD-related gene, Abelson interactor family member 3 (*Abi3)*, is involved in both immune and metabolic regulation. In combination with our previous report demonstrating the importance of *Abi3* in AD-like pathology (Karahan et al., 2021), we have provided evidence to suggest that *Abi3* may serve important roles at the intersection of metabolic, neurodegenerative, and immune regulation.

Our initial interest in the *ABI3* gene was inspired by a human genetics study that identified a rare coding variant within *ABI3* gene locus that is associated with increased risk of AD (Sims et al., 2017). Subsequently, we investigated the impact of *Abi3* deletion on AD neuropathology in the 5xFAD amyloid-β (Aβ) amyloidosis mouse model. We recently reported that deletion of the *Abi3* gene locus significantly exacerbated Aβ pathology and neuroinflammation in these mice (Karahan et al., 2021). In the course of that study, we unexpectedly discovered that *Abi3* knock-out (*Abi3^–/–^*) mice had an obese phenotype.

*Abi3* is predominately expressed in myeloid derived monocytic and microglial immune cells (Zhang et al., 2014; Han et al., 2018). This is relevant in the context of metabolism because recent studies have demonstrated that microglia, in particular microglia within the hypothalamus, affects the progression and onset of obesity (Coats et al., 2017; Valdearcos et al., 2017, 2019; Hill et al., 2018). However, the underlying molecular mechanisms by which these cells contribute to metabolic dysfunction and obesity require further exploration. While much remains to be discovered regarding the cellular functions of *Abi3*, it is suggested to exert its function through its participation in the WASP family verprolin-homologous protein-2 (WAVE2) complex, a regulator of cytoskeletal remodeling (Takenawa and Suetsugu, 2007; Moraes et al., 2017). However, the role of *Abi3* in the context of metabolic regulation and metabolic disease is completely unknown.

In this report, we aim to address this knowledge gap by investigating the impact of *Abi3* deletion on measures of systemic metabolism and obesity. Specifically, we demonstrated that *Abi3* knock-out (*Abi3^–/–^*) mice exhibited an obese phenotype characterized by increased body weight and body fat, as well as impaired glucose tolerance and insulin sensitivity. Further, we determined that *Abi3^–/–^* mice had no changes in food intake but exhibited reduced energy expenditure. Additionally, through RNA-seq analysis, we found that deletion of the *Abi3* gene locus altered gene expression, when controlling for differences in body weight, in the hypothalamus but not adipose tissue. Additionally, microglia number and area were decreased specifically within the mediobasal hypothalamus of *Abi3^–/–^* mice. Altogether, this study is the first to determine the functional importance of the *Abi3* gene locus in the regulation of systemic metabolism and maintenance of healthy weight.

## 2 Material and materials

### 2.1 Animals

The mice utilized in this study were initially generated from a cross between 5XFAD and *Abi3^–/–^* mice; our study utilized the non-transgenic littermates from this cross. The 5xFAD mice were acquired from Jackson Laboratory [MMRRC 34840, B6SJL-Tg (APPSwFlLon, PSEN1* M146L* L286V) 6799Vas/Mmjax)]. The *Abi3^–/–^* mice were acquired from the University of California-Davis MMRRC Mouse Biology Program [C57BL/6N-Abi3tm1.1(KOMP)Vlcg]. The velocigene vector targeted the *Abi3* gene locus on chromosome 11 from 95,842,143 to 95,832,627. Mice were genotyped for presenilin 1 (*PSEN1*) using the primers PSEN1-forward TCATGACTATCCTCCTGGTGG, PS1-reverse CGT TATAGGTTTTAAACACTTCCCC, internal positive control (IPC)-forward CTAGGCCACAGA ATTGAAAGATCT, and IPC-reverse GTAGGTGGAAATTCTAGCATCATCC. Non-transgenic mice were used in this study and genotyped for Abi3 using the following primers: wild-type forward (Wt-F) GGGTCATCTGACAGGTATCTGTTCC, wild-type reverse (Wt-R) CGTGAGTGGGAAGTGTTCTGGTTC, Reg-LacF forward (Reg-Lac-F) ACTTGCTTTAAAAAACCTCCCACA, and Reg-LacF reverse (Reg-Lac-R) GGTTGAGATTGAG AGTCCTTCTGGG. WT-F and WT-R primers produce a band from the Abi3 WT allele, whereas Reg-Lac-F and Reg-Lac-R produce a band from the KO allele. The non-transgenic (no 5xFAD) mice produced from the initial cross were used in this study. Our experiment utilized male mice at 15 months of age. The mice were provided standard housing conditions with *ad libitum* access to food and water.

Ethics Statement- The present study was performed in accordance with our reviewed and approved animal protocol from the Institutional Animal Care and Use Committee of Indiana University School of Medicine.

### 2.2 Tissue preparation

Mice were given Avertin intraperitoneally to induce anesthesia. Mice were then perfused transcardially with PBS. The brains and brown adipose tissue (BAT) were then rapidly removed. For RNA samples, the brains were dissected and immediately frozen in dry ice, along with the brown adipose tissue. For immunofluorescence, brain samples were fixed with 4% paraformaldehyde. The immunofluorescence samples were then passed through a sucrose gradient with 15% sucrose in PBS, followed by 30% sucrose in PBS overnight, followed by embedding and freezing in Fisher Healthcare OCT compound (cat # 23-730-571). All samples were then stored in −80°C prior to their final processing.

### 2.3 Body weight and composition

The mice were weighed on an Ohaus-SCOUT™-SPX622 Portable Precision Balance. Body composition was measured via EchoMRI™ 500. For EchoMRI, mice were guided into a containment tube which restricts movement, and imaging was acquired over a 1–4 min period using a 0.05 T electromagnetic field. Lean mass and fat mass were then calculated from T_1_ and T_2_ relaxing curves via standard algorithms.

### 2.4 Glucose tolerance test

Mice were fasted for 16 h prior to the start of the experiment. The mice then received 1 g/kg glucose by intraperitoneal injection. The tails of the mice were snipped, and blood was then collected at 0, 10, 20, 30, 60, 90, and 120 min. Blood glucose was measured via glucometer at each time point.

### 2.5 Insulin tolerance test

Mice were fasted for 2 h prior to the start of the experiment. The mice then received 0.75 units/kg insulin via intraperitoneal injection. The protocol for measuring blood glucose was identical to GTT, except readings were recorded only at 0, 15, 30, 45, and 60 min.

### 2.6 Metabolic cage analysis

PhenoMaster/LabMaster System (TSE Systems) calorimetry unit was used to measure food and water intake, gas consumption and production, respiratory exchange rate and locomotor activity of the mice. From these recordings, additional measures of metabolism such as energy expenditure and respiratory exchange ratio were generated. Mice were transferred and singly housed in the Phenomaster system, where they were given 3 days to acclimate. Following acclimation, recordings were collected over 84 h.

### 2.7 Total cholesterol assay

Total cholesterol levels were measured in plasma samples using a total cholesterol assay kit (Cell Biolabs, STA-384). Briefly, plasma was added to a reaction mixture that produces a colorimetric probe proportional to the amount of total cholesterol in the sample. The absorbance of the samples and standardized controls were then measured at 562 nm to determine the total amount of cholesterol in each sample, using a BioTek Synergy HTX Multi-Mode Plate Reader. The samples were run at a 1:50 dilution.

### 2.8 Leptin ELISA

Leptin concentration was measured in plasma samples using the U-Plex Mouse Leptin ELISA (Meso Scale Discovery, K1525ZK). Electrochemiluminescence signal was read on a MESO QuickPlex SQ 120. The samples were run at a 1:2 dilution.

### 2.9 RNA extraction

RNA was extracted from the hypothalami and brown adipose tissue via a standard phenol/chloroform extraction using TRIzol reagent (MRC). The concentration and purity of the RNA samples were then assessed via Nanodrop 2000 spectrophotometer. The isolated RNA was then stored at −80°C until further processing.

### 2.10 QuantSeq 3’ mRNA-seq library preparation, sequencing, and processing

Isolated RNA was shipped to Lexogen for library preparation and sequencing. Libraries were prepared manually according to the manufacturer’s instructions using QuantSeq 3’ mRNA-Seq FWD Library Prep Kit as outlined previously (Karahan et al., 2021). Sequencing was performed on a NextSeq 500 instrument with SR75 High Output Kit (Illumina). Seventy-six-base-pair single-end reads were generated. FastQ files were processed based on the workflow outlined in the Lexogen’s user guide for QuantSeq 3’ mRNA-Seq Integrated Data Analysis (Version 015UG108V0310).

### 2.11 Differential gene expression analysis

Gene read count files were imported into RStudio (R v.4.2.0). Differential expression analysis was performed using DESeq2 v.1.36.0 (Love et al., 2014). When body weight was used as a covariate, the weights were first scaled with the scale function from the base R package. Then, the DESeq data set was created using the design argument ∼ Weight + Condition where Weight is the scaled body weight and Condition is the genotype as indicated before. Only genes with total read counts greater than or equal to 5 were included in the model fitting. Log-fold change shrinkage was employed using the lfcShrink function as previously described (Zhu et al., 2018). Differential expressed genes were considered to be genes with an FDR-adjusted (Benjamini-Hochberg) *p*-value ≤ 0.05.

### 2.12 Immunofluorescence

Frozen brains embedded in OCT were coronally sectioned in 30 μm increments, rinsed with PBS, and mounted onto glass slides. Slides were blocked with 2.5% normal goat serum in PBS for 1 h, followed by overnight incubation with Iba1 antibody (1:1,000, Abcam, ab178846) at 4°C. Slides were then incubated with goat anti-rabbit secondary (1:500, Alexa Fluor 568, A11036) for 1.5 h. Slides were then mounted with coverslips using Vectashield Mounting Medium with DAPI (H-1200).

2D images were then acquired with a Leica DMi8 fluorescent microscope at 100× magnification. Images were analyzed by in-house pipelines developed using CellProfiler (Broad Institute) and ImageJ (Schneider et al., 2012; Stirling et al., 2021). For each mouse, three sections from differing anatomical coordinates (separated by 720 μm) were analyzed and the average value from these sections was used. Iba1+ signal and DAPI signal were measured and normalized by the total area of the section. Using CellProfiler, both Iba1+ cell number, cell size, and Iba+ signal area were quantified, as described previously (Valdearcos et al., 2017; Karahan et al., 2021).

### 2.13 Statistical analysis

Statistics were performed with GraphPad Prism 9 software. Comparisons between *Abi3^+/+^* and *Abi3^–/–^*were performed using unpaired *t*-tests. For GTT and ITT, two-way ANOVA with Tukey’s *post-hoc* test was utilized for the time-course graphs. The energy expenditure vs. weight graphs were analyzed using simple linear regression. Data were represented as means ± SEM.

## 3 Results

### 3.1 Increase in body weight and fat mass and impairment of glucose and insulin tolerance in *Abi3^–/–^* mice

**FIGURE 1:**
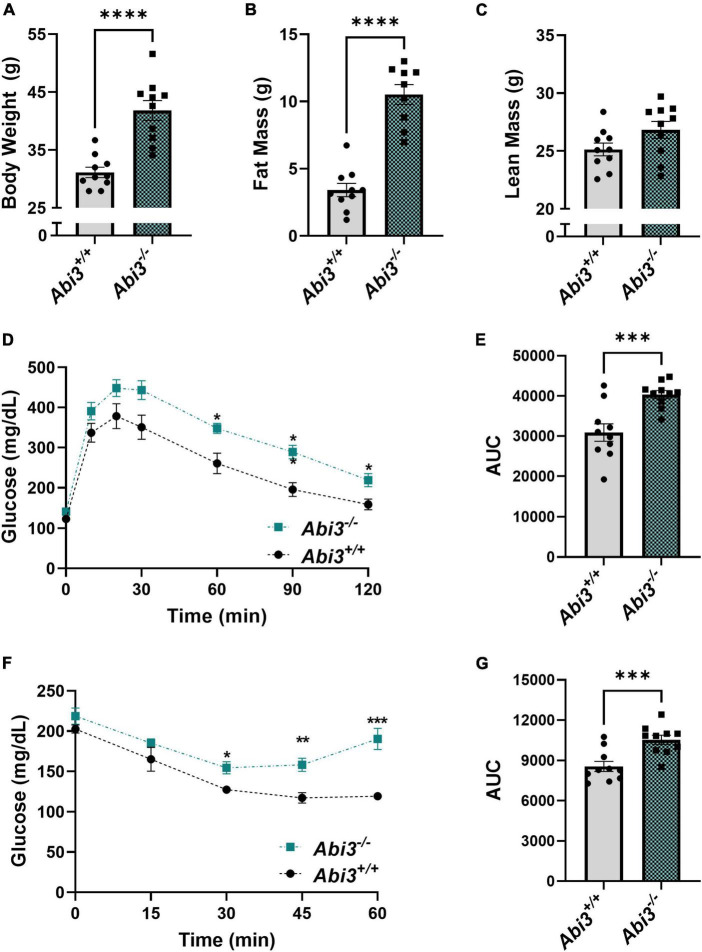
*Abi3^–/–^* mice exhibit increased body weight and fat mass and impaired glucose tolerance and insulin sensitivity. **(A)** Body weight was measured in 15-month-old male *Abi3*^+/+^ (*n* = 10) and *Abi3^–/–^* mice (*n* = 10). **(B,C)** Body composition was measured in these mice using EchoMRI. **(D–G)** Glucose (GTT) and Insulin tolerance tests (ITT) were performed, and the glucose levels of 15-month-old male *Abi3*^+/+^ (*n* = 10) and *Abi3^–/–^* mice (*n* = 10) were recorded over time. **(D)** In the GTT, mice were injected with 1 g/kg glucose following a 16 h fast. Blood glucose (mg/dL) was measured via glucometer at 30, 60, 90, and 120 min post injection. **(E)** The area under the curve for the GTT was calculated. **(F)** In the ITT, mice were injected with 0.75 units/kg insulin following a 2 h fast. Blood glucose (mg/dL) was measured via glucometer at 15, 30, 45, and 60 min post injection. **(G)** The area under the curve for ITT was calculated. Data represent mean ± SEM. For GTT and ITT time-course, 2-way ANOVA with Tukey’s *post-hoc* test was used. For all other comparisons, unpaired *t*-test was used; *p < 0.05, ^**^p < 0.01, ^***^p < 0.001, and ^****^p < 0.0001.

In the course of our AD project, we unexpectedly found that deletion of the *Abi3* gene locus resulted in a dramatic obese phenotype in non-transgenic mice. Because of this unexpected nature, our investigation into the role of ABI3 in metabolism began with already aged mice (15 months). First, we measured the body weight of the *Abi3^+/+^* and *Abi3^–/–^* mice (Figure 1A). Deletion of the *Abi3* gene locus significantly increased body weight, with *Abi3^–/–^* mice weighing an average of 10.70 g, or 32.4%, more than *Abi3^+/+^* mice (Figure 1A). Next, to determine whether this increase in weight was due to increased fat and/or lean mass, we performed EchoMRI body composition analysis on these mice (Figures 1B, C). Similar to body weight, fat mass was significantly increased in *Abi3^–/–^* mice. Specifically, *Abi3^–/–^* mice had more fat mass than *Abi3^+/+^* mice by an average of 7.11 g, or 208.4% (Figure 1B). Lean mass, however, was not significantly altered in *Abi3^–/–^* mice (Figure 1C).

To further understand the impact of the loss of ABI3 function on systemic glucose regulation, we performed glucose tolerance (GTT) and insulin tolerance (ITT) tests (Figures 1D–G). In the GTT, *Abi3^–/–^* mice exhibited significantly elevated blood glucose levels as compared to *Abi3^+/+^* mice at 60, 90, and 120 min following glucose administration (Figure 1D). Similarly, area under the curve analysis of GTT data revealed that *Abi3^–/–^* mice had significantly greater area than *Abi3^+/+^* mice (Figure 1E). These data indicate that *Abi3^–/–^* mice had impaired glucose tolerance relative to control. In the ITT, *Abi3^–/–^* mice had significantly elevated blood glucose levels at 30, 45, and 60 min, indicative of insulin resistance, as compared to *Abi3^+/+^* mice (Figure 1F). Area under the curve analysis of ITT data revealed that *Abi3^–/–^* mice had significantly greater area than *Abi3^+/+^* mice, indicating that *Abi3^–/–^* mice had impaired insulin response relative to control (Figure 1G).

### 3.2 Energy expenditure during light cycle is impaired, while food intake is unchanged, in *Abi3^–/–^* mice

**FIGURE 2:**
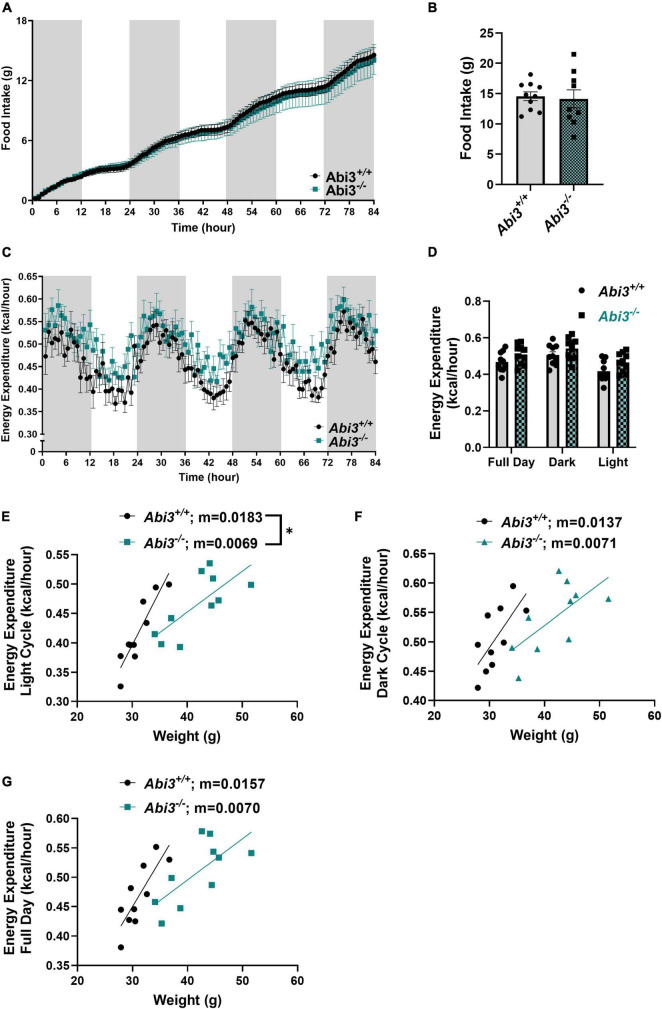
*Abi3^– /–^* mice exhibit impaired energy expenditure and unaltered food intake. 15-month-old male *Abi3*^+/+^ (*n* = 10) and *Abi3^– /–^* mice (*n* = 10) were singly housed in metabolic cages and **(A,B)** food intake and **(C,D)** energy expenditure were recorded over 84 h. For time course graphs **(A,C)**, the shaded portions represent night hours (dark cycle) and the unshaded portions represent daytime (light cycle). **(A)** Food intake over time and **(B)** overall total food intake were recorded. **(C)** Energy expenditure over time and **(D)** average energy expenditure were measured. **(D)** Average energy expenditure was calculated for the daytime/light hours (light cycle), night/dark hours (dark cycle), and the entire 84 h (full day). **(E–G)** Subsequent linear regression analysis of average energy expenditure vs. weight was performed to assess the relationship between lean mass and energy expenditure. Regressions were performed for **(E)** light cycle, **(F)** dark cycle, and **(G)** full day. Data represent mean ± SEM. Unpaired *t*-test, simple linear regression; *p < 0.05.

To determine if the obese phenotype was secondary to differences in food intake, energy expenditure, or a combination of these factors, we performed comprehensive metabolic phenotyping and indirect calorimetry, via metabolic cage, on the *Abi3^+/+^* and *Abi3^–/–^* mice (Figure 2). First, we measured the food consumed during the 84-h testing period within the Phenomaster system. The deletion of *Abi3* gene locus did not alter food intake between groups (Figures 2A, B). Next, we evaluated energy expenditure from the calorimetry experiment during the light, dark, and combined full day cycles. There was no gross change in average energy expenditure between the genotypes (Figures 2C, D). However, further regression analysis comparing the relationship of energy expenditure vs. weight revealed that *Abi3^–/–^* mice had significantly lower slope than *Abi3^+/+^* mice during the light cycle, indicative of impaired energy expenditure (Figure 2E). No significant differences in slope were detected between *Abi3^–/–^* and *Abi3^+/+^* mice for the dark cycle or full day regression analyses (Figures 2F, G). Additionally, no differences in respiratory exchange ratio, a measure of carbohydrate vs. lipid fuel utilization, were observed between the *Abi3^+/+^* and *Abi3^–/–^* mice (Supplementary Figure 1B). Finally, to determine if differences in energy expenditure were secondary to changes in overall movement, we evaluated the total and average movement of the *Abi3^+/+^* and *Abi3^–/–^* mice during the calorimetry experiment (Supplementary Figure 2). *Abi3* genotype status had no impact on total or average movement counts (Supplementary Figure 2C).

### 3.3 Total cholesterol and leptin concentration are increased in the plasma of *Abi3^–/–^* mice

In order to further evaluate the metabolic consequences observed in *Abi3^–/–^* mice, we performed cholesterol and leptin measurements on the plasma from *Abi3^+/+^* and *Abi3^–/–^* mice. Using a colorimetric total cholesterol assay, we found that *Abi3^–/–^* mice had significantly elevated cholesterol levels (Supplementary Figure 3). Then, we performed a leptin ELISA on the plasma from these mice and similarly observed that *Abi3^–/–^* mice had significantly elevated leptin concentration (Supplementary Figure 3). Elevated leptin and cholesterol levels are characteristic of obesity, and therefore these results further demonstrate the extent of the obese phenotype observed in *Abi3^–/–^* mice (Klop et al., 2013; Obradovic et al., 2021).

### 3.4 *Abi3* locus deletion alters the transcriptome, independent of differences in weight, in the hypothalamus but not in brown adipose tissue

**FIGURE 3:**
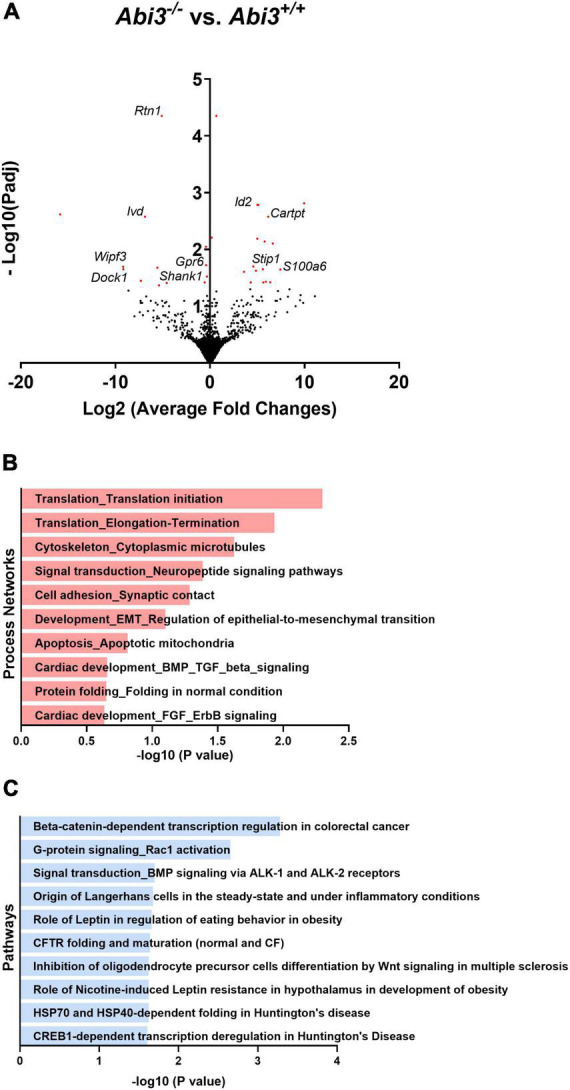
Deletion of *Abi3* gene locus, independent of differences in body weight, alters the transcriptome within the hypothalamus. Bulk RNA sequencing was performed with the hypothalami of 15-month-old male *Abi3*^+/+^ (*n* = 4) and *Abi3^– /–^* mice (*n* = 4). Differentially expressed genes (DEGs), defined by an adjusted *p*-value < 0.05, were then identified. **(A)** Volcano plot demonstrating the DEGs from the hypothalami of *Abi3^– /–^* vs. *Abi3*^+/+^mice. The red points represent differentially expressed genes. **(B,C)** The DEGs were used to perform **(B)** process network and **(C)** pathway analysis using the Metacore software. The significantly enriched **(B)** process networks and **(C)** pathways are listed. For additional information on **(B,C)** the Metacore figures, see Supplementary Table 1.

To gain insight into the potential pathways that are regulated by *Abi3* gene locus deletion, we evaluated the gene expression profile of tissues central to body weight and energy expenditure regulation by performing bulk RNA-seq on the hypothalami and brown adipose tissue of *Abi3^+/+^* and *Abi3^–/–^* mice. To isolate changes that were due to the absence of *Abi3* itself, rather than secondary to *Abi3* dependent differences in body weight, we performed weight-covariate controlled analysis of the RNA-seq data. For our analysis, differentially expressed genes (DEGs) were defined by a cut off of adjusted *p*-values less than 0.05. Within the brown adipose tissue, no DEGs were identified between *Abi3^–/–^* vs. *Abi3^+/+^* mice (Supplementary Figure 4). Within the hypothalamus, however, 31 weight covariate controlled DEGs were identified, including *Shank1* and *Cartpt* (Figure 3A).

To determine the potential biologic relevance of these DEGs identified within the hypothalamus, we performed secondary process network and pathway analyses using the Metacore software. The DEGs identified in *Abi3^–/–^* mice were significantly enriched in biologic processes including cytoskeletal regulation, synaptic cellular adhesion, and neuropeptide signaling, among others (Figure 3B). Further, these DEGs were significantly enriched in pathways including G-protein/Rac1 signaling and leptin regulation, among others (Figure 3C).

### 3.5 Microglia number and area were decreased in the mediobasal hypothalamus, but not other brain areas in *Abi3^–/–^* mice

**FIGURE 4:**
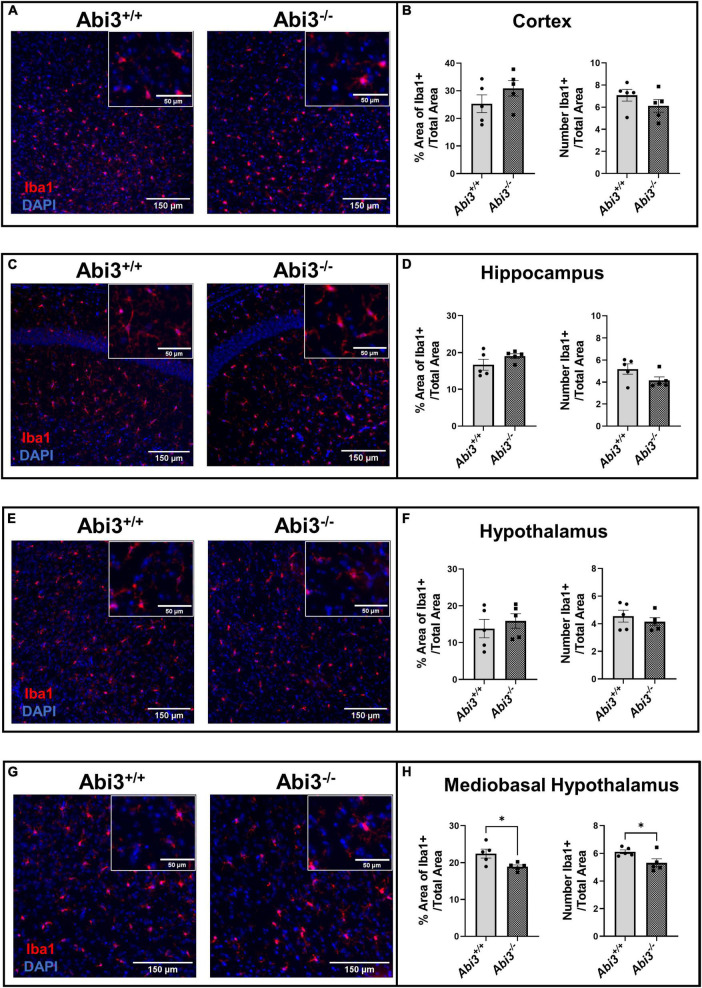
Deletion of *Abi3* gene locus reduces microglia number and area within the mediobasal hypothalamus, but not other brain regions. Coronal brain sections from 15-month-old male *Abi3*^+/+^ (*n* = 5) and *Abi3^– /–^* mice (*n* = 5) were stained with Iba1+ (microglia marker) and DAPI (nuclear counterstain). **(A,C,E,G)** Representative images, for each brain region, show Iba1 + signal in red and DAPI in blue. **(B,D,F,H)** The number of microglia and area occupied by microglia were quantified across these brain regions. The area and number of Iba1 + signal was measured in the **(A,B)** cortex, **(C,D)** hippocampus, **(E,F)** whole hypothalamus, and **(G,H)** mediobasal hypothalamus. Data represent mean ± SEM. Unpaired *t*-test; *p < 0.05.

Because *Abi3* is predominantly expressed by microglia within the CNS, we performed Iba1 immunofluorescence on brain sections from *Abi3^–/–^* and *Abi3^+/+^* mice. Specifically, we assessed the changes in microglia number and area throughout multiple CNS regions (Figure 4). The total number or area of Iba1+ cells were not altered in the cortex or hippocampus of *Abi3^–/–^* mice (Figures 4A–D). Iba1+ cell size was increased only in the hippocampus, but not other brain regions of *Abi3^–/–^* mice (Supplementary Figure 5). Further, there was no significant difference in Iba1+ cell number or area when evaluating the entire hypothalamus (Figures 4E, F). However, within the mediobasal subregion of the hypothalamus, a region critical to the regulation of energy balance, *Abi3^–/–^* mice showed significantly lower number and area of Iba1+ cells (Figures 4G, H). The mediobasal hypothalamus is an area that is central to the regulation of systemic metabolism, and it is postulated that microglia play a key role in that regulation. Therefore, the change in microglia number could indicate that the loss of *Abi3* might induce obesity through altered microglia function within the mediobasal hypothalamus.

## 4 Discussion

In this report, we demonstrated that *Abi3^–/–^* mice had significantly elevated body and fat mass, and reduced energy expenditure. Importantly, these mice had reduced microglia number and area within the mediobasal hypothalamus, the brain region critical to the regulation of energy balance. Altogether, our data suggest that the functions of ABI3 are not only important in the response to neurodegenerative pathology (Karahan et al., 2021) but also in the regulation of systemic metabolism.

Due to the unexpected nature of our initial discovery regarding the impact of *Abi3* deletion on body weight, our original observation of the obese phenotype did not occur until the mice were 15 months old. In these mice, we found that body weight and fat mass were significantly increased in *Abi3^–/–^* mice, while lean mass was not altered. These findings indicated that the increased weight was driven predominantly by increased fat mass, which is indicative of an obese phenotype. Furthermore, we found that *Abi3^–/–^* mice had impairments in both glucose tolerance and insulin sensitivity. This likely suggests that the obesity in *Abi3^–/–^* mice induced impairments to systemic glucose regulation, as is typically observed in obese mice (King, 2012). Additionally, as these findings were observed in aged mice, it is possible that the obese phenotype is an age dependent phenomenon. Future study can help elucidate whether deletion of *Abi3* at earlier time points induces similar metabolic effects. Additionally, only male mice were available for this investigation because female mice were already used for another project. Therefore, future studies will be necessary to determine the potential impact of sex dependent differences on the obese phenotype.

We further investigated whether the difference in weight was driven by increased food intake, decreased energy expenditure, or a combination of both. The food intake and the overall movement of the mice did not change between the genotypes. However, *Abi3^–/–^* mice had significantly reduced slope on the regression of energy expenditure vs. body weight during the light cycle but not the dark cycle. This indicates that *Abi3^–/–^* mice have reduced energy expenditure during their more inactive hours relative to *Abi3^+/+^* mice. Overall, these findings suggest that the loss of ABI3 function may drive obesity through impairments to energy expenditure. It is important, however, to note that these findings were observed in mice that were already obese. Therefore, it is possible that there might be additional mechanisms driving the obese phenotype in *Abi3^–/–^* mice. Critically, however, the regression analysis addressed the potential confounding effects of differing body weight (Tschöp et al., 2011), thus isolating the direct effect of the deletion of *Abi3* on energy expenditure.

Energy expenditure in mice is predominantly regulated by the hypothalamus, centrally, and largely by thermogenic brown adipose tissue (BAT), peripherally (Varela and Horvath, 2012; Harms and Seale, 2013; Schneeberger et al., 2014). Since we observed that *Abi3^–/–^*mice exhibited impaired energy expenditure, we performed bulk RNA-seq on these tissues from *Abi3^+/+^* and *Abi3^–/–^* mice. In an attempt to isolate the direct effect of *Abi3* locus deletion on gene expression within these tissues, we performed a weight-covariate controlled analysis. This approach did not identify any DEGs within the BAT but did identify DEGs within the hypothalamus. This suggests that the deletion of *Abi3* gene locus itself, as opposed to *Abi3* dependent differences in body weight, induced changes to gene expression within the hypothalamus but not within BAT. This could suggest that hypothalamus, not BAT, triggered the initial dysfunction that subsequently drives the impairment to energy expenditure. Furthermore, our pathway analysis indicated that the hypothalamic DEGs were enriched in biologic processes including cellular adhesion at the synapse, energy balance related neuropeptide signaling (*Cartpt*) and cytoskeletal remodeling (*Shank1*). The enrichment of the cytoskeletal related processes matches with the previously reported functions of ABI3. Specifically, ABI3 has been reported to be participant in cellular processes that require remodeling of the actin cytoskeleton, including cellular migration and membrane protrusion (Sekino et al., 2015; Karahan et al., 2021). As *Abi3* is predominantly expressed by microglia within the CNS, our transcriptomic analyses suggests that the deletion of *Abi3* is impacting the function of microglia within the hypothalamus. Further, microglia are highly dynamic cells that are constantly surveying their microenvironment and communicating with other CNS cells, both of which are functions that require cytoskeletal remodeling (Uhlemann et al., 2016; Prinz et al., 2019). Therefore, these sequencing data might point toward impaired cytoskeletal remodeling, and subsequently an impaired ability of microglia to perform their homeostatic functions, as a contributing factor to the induction of the obese phenotype. However, future investigation will be required to comprehensively test this hypothesis.

Importantly, altered microglia function within the hypothalamus has been connected to the maintenance of body weight and obesity (Valdearcos et al., 2014, 2017; Andre et al., 2017; Dorfman et al., 2017; Gao et al., 2017; Rosin et al., 2018; De Luca et al., 2019; Kim et al., 2019; Campolim et al., 2020). Multiple studies have demonstrated that microglia number increases within the mediobasal hypothalamus during diet induced obesity (Thaler et al., 2012; de Kloet et al., 2014; Valdearcos et al., 2014; Schur et al., 2015; Waise et al., 2015; Baufeld et al., 2016; Guillemot-Legris et al., 2016). Further, it has been demonstrated that reducing this dietary induced microgliosis can mitigate weight gain (Andre et al., 2017; Valdearcos et al., 2017; Kim et al., 2019). It has also been reported that genetic or pharmacologic treatments that increase microgliosis within the mediobasal hypothalamus are capable of inducing obesity even in the absence of dietary challenge (Valdearcos et al., 2017; Campolim et al., 2020). Conversely, a recent study demonstrated that by deleting a gene specifically within microglia, the number of microglia within the mediobasal hypothalamus decreased but diet induced obesity was exacerbated (Gao et al., 2017). Given the importance of microglia within the mediobasal hypothalamus to obesity, in conjunction with our sequencing analysis, we evaluated whether *Abi3^–/–^* mice had altered microglia number within the mediobasal hypothalamus or any other CNS region. Intriguingly, we found that microglia number and area were decreased specifically within the mediobasal hypothalamus, but not in other areas, of *Abi3^–/–^* mice compared to *Abi3^+/+^*. We are not the first to report a decrease in the number of mediobasal hypothalamic microglia in the setting of impaired body weight regulation; however, to the best of our knowledge, we are the first to report such findings in the absence of dietary intervention.

Our data contribute to the growing concept that an appropriate level of microglia activity within the hypothalamus is necessary to maintain proper control of energy balance (Valdearcos et al., 2019). It appears that both hyperreactivity and the reduced reactivity of hypothalamic microglia may lead to dysfunctional regulation of systemic metabolism. Intriguingly, a similar paradigm regarding microglia activity has become well-established in the neurodegenerative disease field (Hickman et al., 2018). Currently, however, little is known about the exact cellular mechanisms by which hypothalamic microglia modulate energy balance. It has been postulated that microglia regulate control of energy balance via the modulation of neuronal signaling within the hypothalamus through microglia-neuron interactions (Folick et al., 2022). Recent studies have shown that both increased and decreased microglial inflammatory activity within the mediobasal hypothalamus can disrupt leptin/melanocortin related neuronal signaling, a pathway that is central to the regulation of energy balance (Gao et al., 2017; Valdearcos et al., 2017; Kim et al., 2019). Further, it has been suggested that microglia may directly alter the organization of melanocortin system neuronal synapses (Horvath et al., 2010; Kim et al., 2019).

In the context of this study, it is possible that the deletion of the *Abi3* gene locus impaired the ability of microglia to perform the complex functions that are necessary to regulate energy balance (Valdearcos et al., 2019; De Luca et al., 2020). This possibility seems feasible when considering the importance of dynamic cytoskeletal remodeling for microglia to perform their basic functions (migration, surveillance, phagocytosis, etc.), along with the reported importance of ABI3 in processes that require cytoskeletal remodeling such as migration and phagocytosis (Sekino et al., 2015; Karahan et al., 2021). However, future study will be necessary to uncover the specific functional impairment in microglia that led to impaired regulation of energy balance in *Abi3^–/–^* mice. Additionally, while our data are suggestive of a CNS driven disruption of energy balance, it remains possible that peripherally driven dysfunction could partly underly the obese phenotype. Future studies could utilize microglial specific inducible knock-out of *Abi3* to address this limitation more definitely.

Overall, this report provides a novel, initial exploration into the role of *Abi3* in the regulation of systemic metabolism, opening the door to future investigation aimed at uncovering how the manipulation of ABI3-related function can modulate metabolic disease states. Furthermore, our study has now demonstrated how deletion of *Abi3* gene locus can have dramatic impacts on the seemingly distinct disease states of obesity and neurodegeneration, likely due to disruptions in microglia functions (Karahan et al., 2021). Together, our study helps to address the critical knowledge gap regarding the specific molecular drivers that may jointly underly the microglia dysfunction observed in metabolic and neurodegenerative disease.

## Data availability statement

The datasets presented in this study can be found in online repositories. The names of the repository/repositories and accession number(s) can be found below: https://www.ncbi.nlm.nih.gov/geo/, GSE212299.

## Ethics statement

The animal study was reviewed and approved by the IUPUI Animal Care and Use Committee Indiana University School of Medicine at Indianapolis.

## Author contributions

DS, HK, and JK created the overall study design and interpreted the findings. DS, HK, BM, and YM performed the experiments. DS performed the metabolic cage data analysis. HW and HK performed the bioinformatic analyses. DS and MA-A analyzed the immunofluorescence data. DS, HK, HW, and JK wrote the manuscript. All authors contributed to the article and approved the submitted version.
