# Supplementary material for: Deletion of the Alzheimer’s disease risk gene Abi3 locus results in obesity and systemic metabolic disruption in mice
Source: Front Aging Neurosci. 2022 Dec 22;14:1035572. doi: 10.3389/fnagi.2022.1035572 (PMC9813750; doi:10.3389/fnagi.2022.1035572)
Supplement: Supplementary file 1 [file Presentation_1.pptx]

## Slide 1
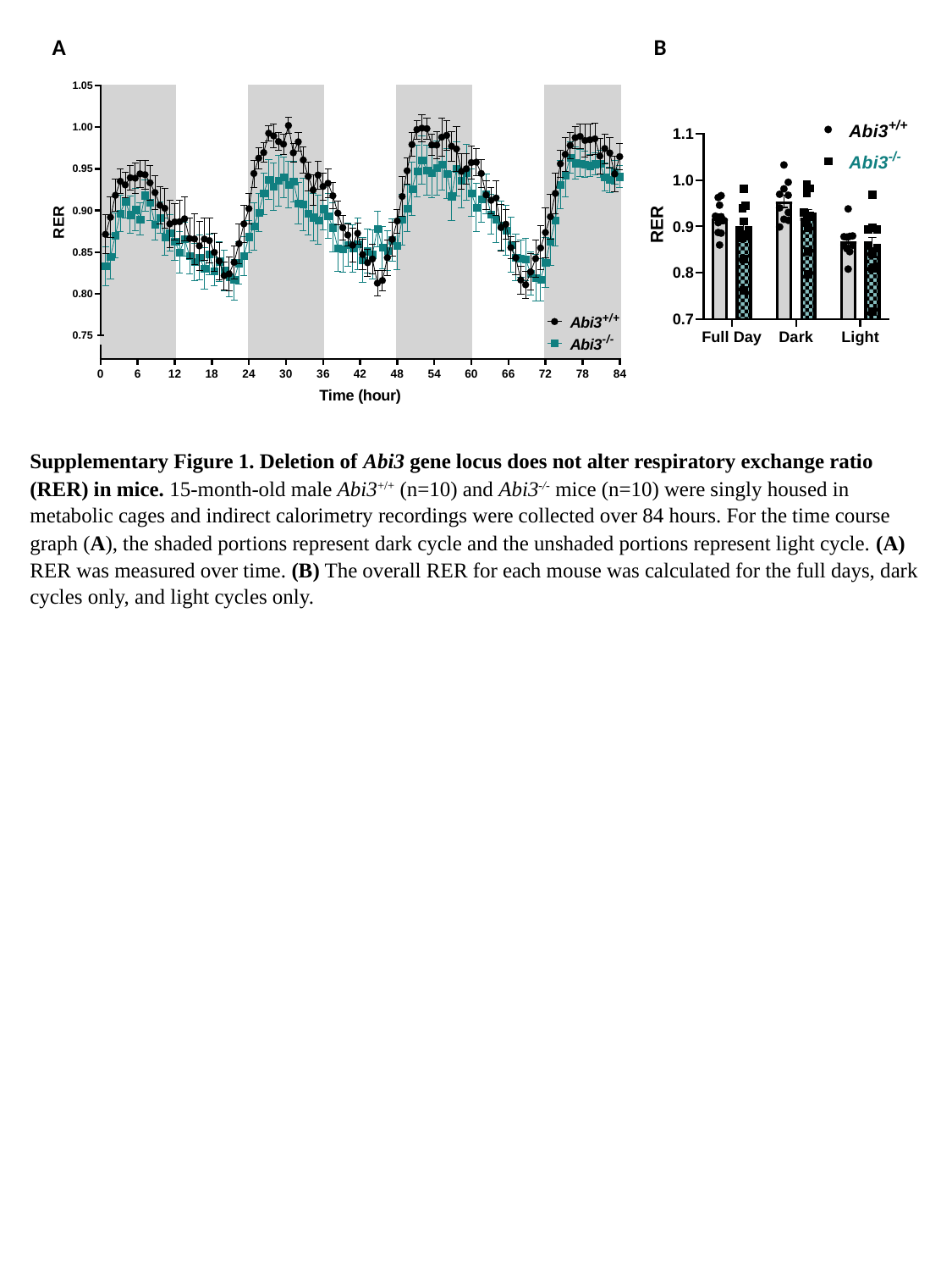

A
B
0
Supplementary Figure 1. Deletion of Abi3 gene locus does not alter respiratory exchange ratio (RER) in mice. 15-month-old male Abi3+/+ (n=10) and Abi3-/- mice (n=10) were singly housed in metabolic cages and indirect calorimetry recordings were collected over 84 hours. For the time course graph (A), the shaded portions represent dark cycle and the unshaded portions represent light cycle. (A) RER was measured over time. (B) The overall RER for each mouse was calculated for the full days, dark cycles only, and light cycles only.

## Slide 2
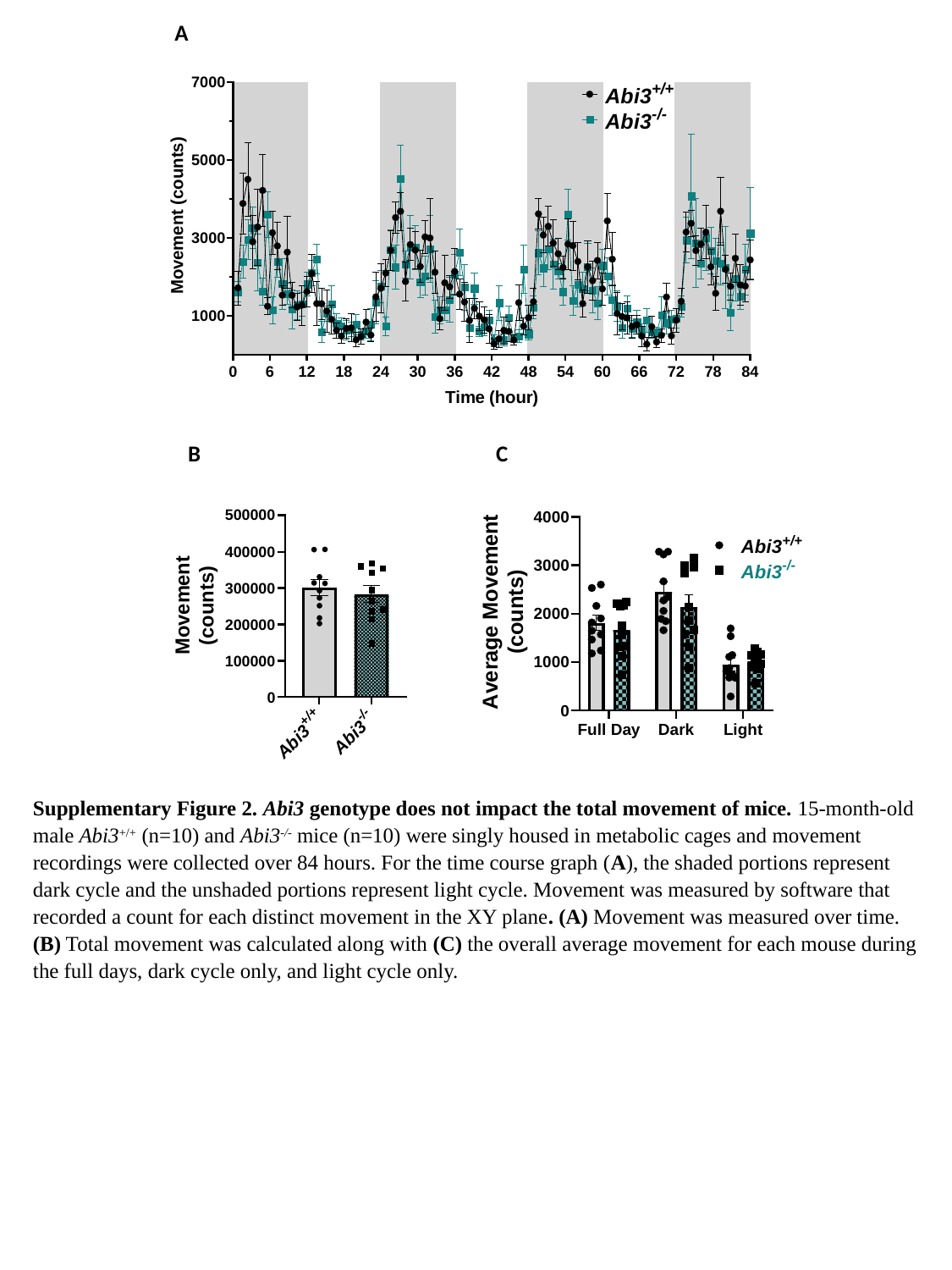

A
0
B
C
Supplementary Figure 2. Abi3 genotype does not impact the total movement of mice. 15-month-old male Abi3+/+ (n=10) and Abi3-/- mice (n=10) were singly housed in metabolic cages and movement recordings were collected over 84 hours. For the time course graph (A), the shaded portions represent dark cycle and the unshaded portions represent light cycle. Movement was measured by software that recorded a count for each distinct movement in the XY plane. (A) Movement was measured over time. (B) Total movement was calculated along with (C) the overall average movement for each mouse during the full days, dark cycle only, and light cycle only.

## Slide 3
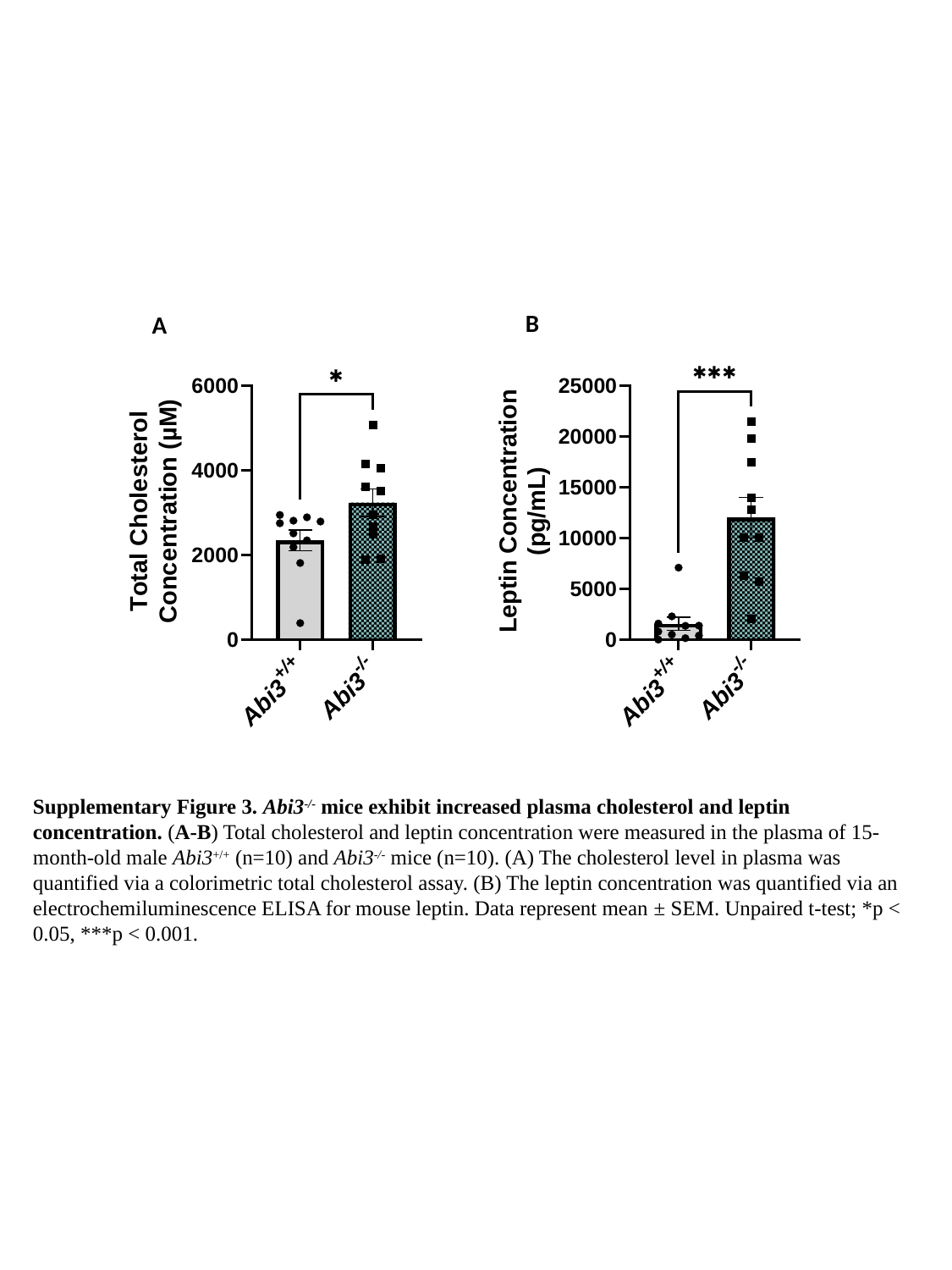

B
A
Supplementary Figure 3. Abi3-/- mice exhibit increased plasma cholesterol and leptin concentration. (A-B) Total cholesterol and leptin concentration were measured in the plasma of 15-month-old male Abi3+/+ (n=10) and Abi3-/- mice (n=10). (A) The cholesterol level in plasma was quantified via a colorimetric total cholesterol assay. (B) The leptin concentration was quantified via an electrochemiluminescence ELISA for mouse leptin. Data represent mean ± SEM. Unpaired t-test; *p < 0.05, ***p < 0.001.

## Slide 4
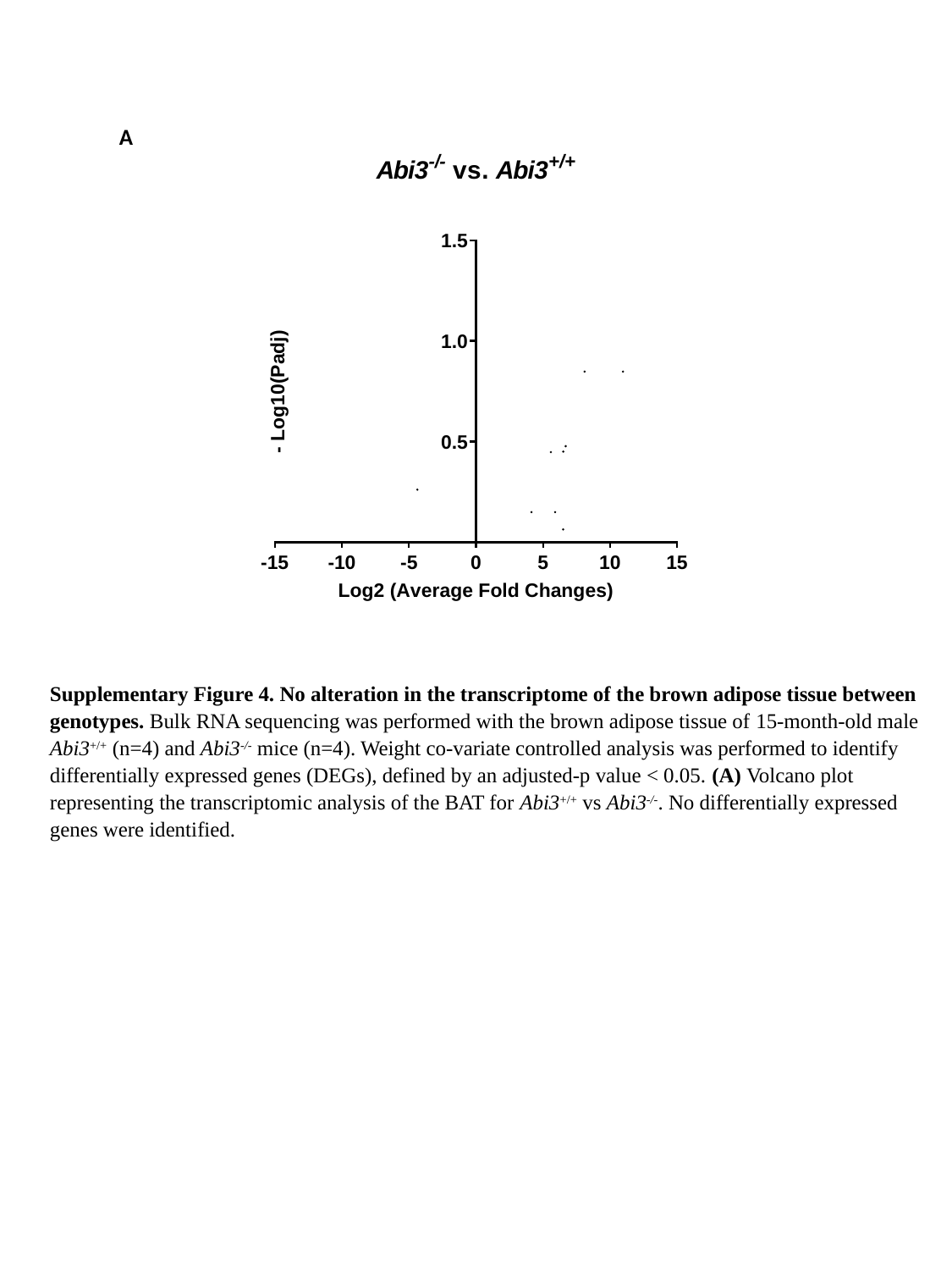

A
0
Supplementary Figure 4. No alteration in the transcriptome of the brown adipose tissue between genotypes. Bulk RNA sequencing was performed with the brown adipose tissue of 15-month-old male Abi3+/+ (n=4) and Abi3-/- mice (n=4). Weight co-variate controlled analysis was performed to identify differentially expressed genes (DEGs), defined by an adjusted-p value < 0.05. (A) Volcano plot representing the transcriptomic analysis of the BAT for Abi3+/+ vs Abi3-/-. No differentially expressed genes were identified.

## Slide 5
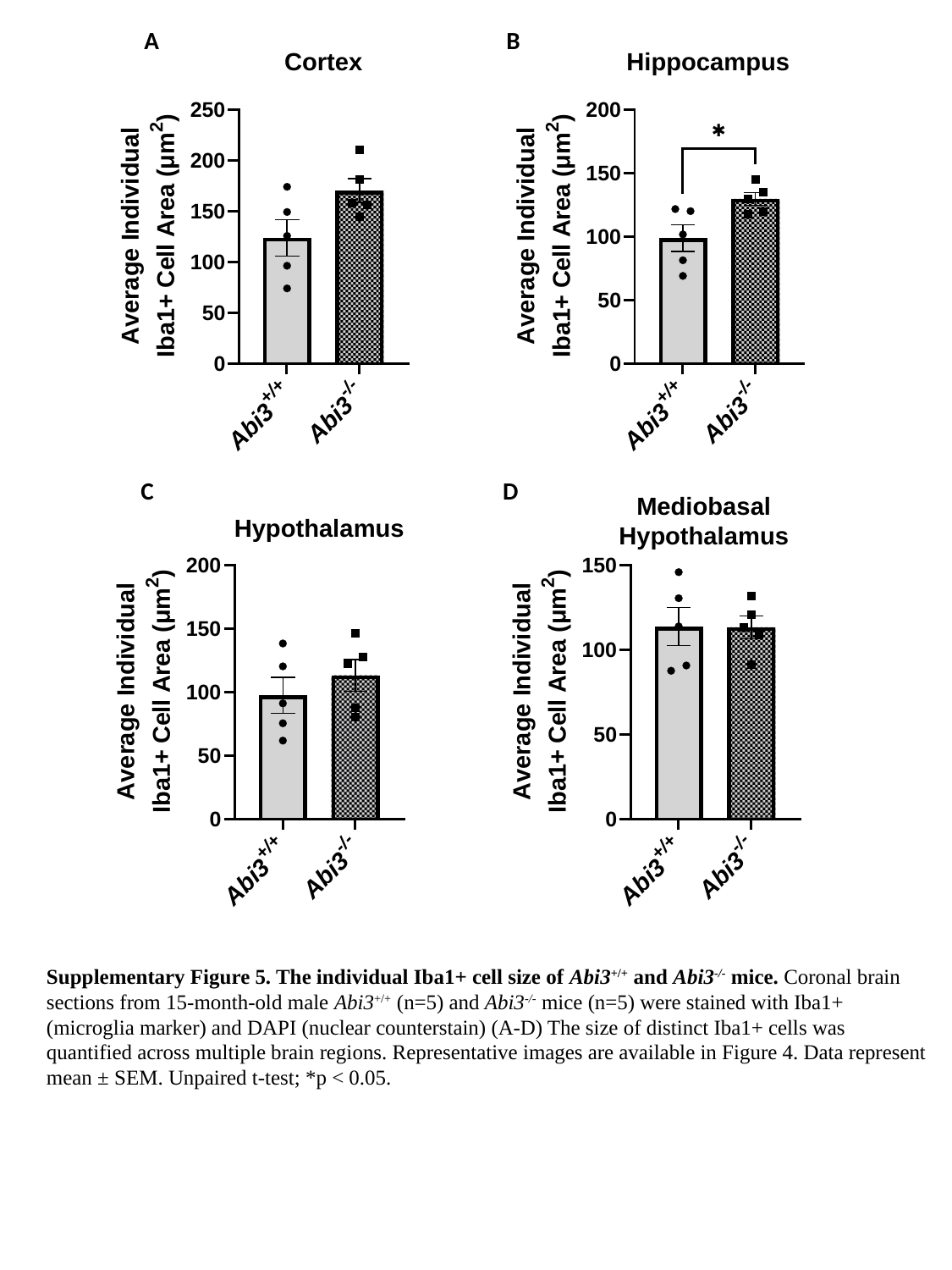

A
B
Hippocampus
Cortex
C
D
Mediobasal Hypothalamus
Hypothalamus
Supplementary Figure 5. The individual Iba1+ cell size of Abi3+/+ and Abi3-/- mice. Coronal brain sections from 15-month-old male Abi3+/+ (n=5) and Abi3-/- mice (n=5) were stained with Iba1+ (microglia marker) and DAPI (nuclear counterstain) (A-D) The size of distinct Iba1+ cells was quantified across multiple brain regions. Representative images are available in Figure 4. Data represent mean ± SEM. Unpaired t-test; *p < 0.05.

## Slide 6
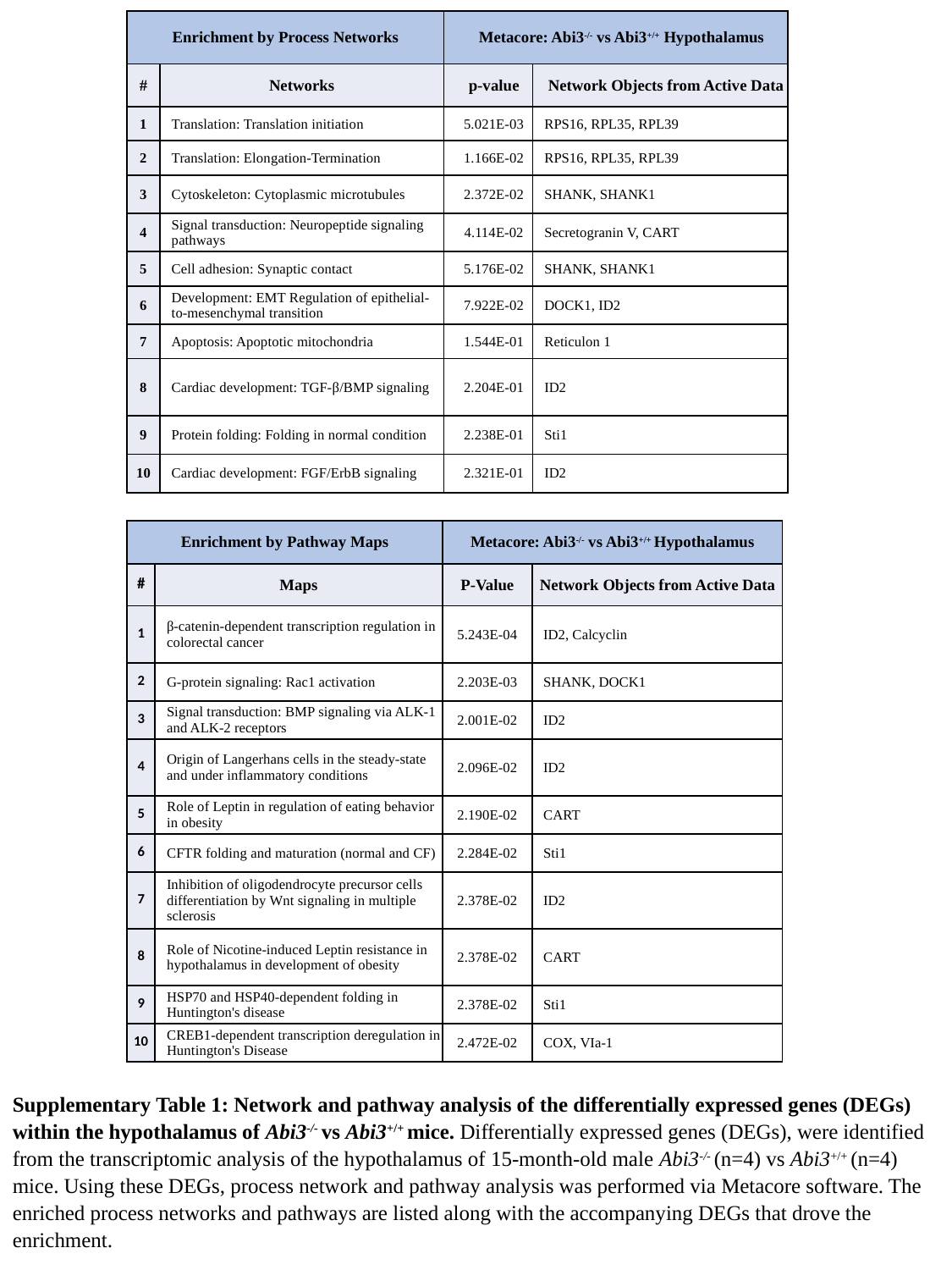

| Enrichment by Process Networks | | Metacore: Abi3-/- vs Abi3+/+ Hypothalamus | |
| --- | --- | --- | --- |
| # | Networks | p-value | Network Objects from Active Data |
| 1 | Translation: Translation initiation | 5.021E-03 | RPS16, RPL35, RPL39 |
| 2 | Translation: Elongation-Termination | 1.166E-02 | RPS16, RPL35, RPL39 |
| 3 | Cytoskeleton: Cytoplasmic microtubules | 2.372E-02 | SHANK, SHANK1 |
| 4 | Signal transduction: Neuropeptide signaling pathways | 4.114E-02 | Secretogranin V, CART |
| 5 | Cell adhesion: Synaptic contact | 5.176E-02 | SHANK, SHANK1 |
| 6 | Development: EMT Regulation of epithelial-to-mesenchymal transition | 7.922E-02 | DOCK1, ID2 |
| 7 | Apoptosis: Apoptotic mitochondria | 1.544E-01 | Reticulon 1 |
| 8 | Cardiac development: TGF-β/BMP signaling | 2.204E-01 | ID2 |
| 9 | Protein folding: Folding in normal condition | 2.238E-01 | Sti1 |
| 10 | Cardiac development: FGF/ErbB signaling | 2.321E-01 | ID2 |
| Enrichment by Pathway Maps | | Metacore: Abi3-/- vs Abi3+/+ Hypothalamus | |
| --- | --- | --- | --- |
| # | Maps | P-Value | Network Objects from Active Data |
| 1 | β-catenin-dependent transcription regulation in colorectal cancer | 5.243E-04 | ID2, Calcyclin |
| 2 | G-protein signaling: Rac1 activation | 2.203E-03 | SHANK, DOCK1 |
| 3 | Signal transduction: BMP signaling via ALK-1 and ALK-2 receptors | 2.001E-02 | ID2 |
| 4 | Origin of Langerhans cells in the steady-state and under inflammatory conditions | 2.096E-02 | ID2 |
| 5 | Role of Leptin in regulation of eating behavior in obesity | 2.190E-02 | CART |
| 6 | CFTR folding and maturation (normal and CF) | 2.284E-02 | Sti1 |
| 7 | Inhibition of oligodendrocyte precursor cells differentiation by Wnt signaling in multiple sclerosis | 2.378E-02 | ID2 |
| 8 | Role of Nicotine-induced Leptin resistance in hypothalamus in development of obesity | 2.378E-02 | CART |
| 9 | HSP70 and HSP40-dependent folding in Huntington's disease | 2.378E-02 | Sti1 |
| 10 | CREB1-dependent transcription deregulation in Huntington's Disease | 2.472E-02 | COX, VIa-1 |
Supplementary Table 1: Network and pathway analysis of the differentially expressed genes (DEGs) within the hypothalamus of Abi3-/- vs Abi3+/+ mice. Differentially expressed genes (DEGs), were identified from the transcriptomic analysis of the hypothalamus of 15-month-old male Abi3-/- (n=4) vs Abi3+/+ (n=4) mice. Using these DEGs, process network and pathway analysis was performed via Metacore software. The enriched process networks and pathways are listed along with the accompanying DEGs that drove the enrichment.
